# Genotoxic Activity of *Cerastes cerastes* Viper Venom Using Alkaline Single-Cell Gel Electrophoresis

**DOI:** 10.3390/biology15171485

**Published:** 2026-09-02

**Authors:** Zineb Ouhemmou, Nouhaila Benbaak, Zahraa Laouija, Soukaina Behlaouane, Nouhaila El Fenni, Mouad Mkamel, Mounia Cherki, Mehdi Karkouri, Fatima Chgoury

**Affiliations:** 1 Venoms and Toxins Laboratory, Pasteur Institute of Morocco, Casablanca 20360, Morocco; zineb.ouhemmou-etu@etu.univh2c.ma (Z.O.); nohaila.benbaak-etu@etu.univh2c.ma (N.B.); zahraa.laouija-etu@etu.univh2c.ma (Z.L.); soukaina.behlaouane-etu@etu.univh2c.ma (S.B.); nouhaila.elfenni-etu@etu.univh2c.ma (N.E.F.); 2 Health, Environment and Biotechnology Laboratory, Faculty of Sciences Aïn Chock, Hassan II University of Casablanca, Casablanca 20000, Morocco; cherkimoun@gmail.com; 3 Biology and Health Laboratory, Faculty of Sciences Ben M’sick, Hassan II University of Casablanca, Casablanca 20700, Morocco; mouad.mkamel@univh2c.ma; 4 Laboratory of Ecology and Environment, Faculty of Sciences Ben M’Sik, Hassan II University of Casablanca, Casablanca 20700, Morocco; 5 Department of Cellular and Molecular Pathology, Faculty of Medicine and Pharmacy, Hassan II University of Casablanca, Casablanca 20250, Morocco; m.karkouri@chucasa.ma

**Keywords:** genotoxicity, cytotoxicity, *Cerastes cerastes* venom, comet assay, alkaline single-cell gel electrophoresis, oxidative stress

## Abstract

In Morocco, *Cerastes cerastes* snake is responsible for numerous cases of human envenomation, leading to both local and systemic pathophysiological disturbances. This study provides a first preliminary evaluation of cytotoxic and genotoxic effects of its venom on genomic material (Deoxyribonucleotide acid: DNA). The venom protein profile was characterized using sodium dodecyl sulfate–polyacrylamide gel electrophoresis, and its median lethal dose (LD_50_) was determined by intraperitoneal injection in mice. Genotoxicity was assessed using the alkaline comet assay (single-cell gel electrophoresis) on peripheral blood cells following in vitro and in vivo venom treatments with sampling performed between 1 and 24 h after exposure. Concurrently, cell death was evaluated using the trypan blue exclusion assay as an indicator of venom-induced cytotoxicity. DNA damage parameters, including percentage of DNA in the head, tail and tail length, were quantified using *OpenComet* software. The venom exhibited a protein-rich composition and an LD_50_ of 34.64 µg/mouse. The comet assay revealed statistically significant DNA damage compared to the control group. Partial to complete DNA repair was noted after 24 h depending on the administered dose. These findings confirm those obtained with cell death assay. All results indicate that Moroccan *C. cerastes* venom induces transient DNA damage, potentially associated with oxidative stress mechanisms leading to cytotoxicity. It is primordial to characterize target venom-molecules exhibiting genetic failure in order to find an adequate therapeutic approach.

## Simple Summary

Snakebite envenomation caused by *Cerastes cerastes* represents an important public health concern in Morocco, where victims may develop severe local and systemic complications. Although the toxic effects of snake venoms are well documented, their potential impact on genetic material remains poorly explored. In this study, we investigated the genotoxic effects of *C. cerastes* venom using the alkaline comet assay following both in vitro and in vivo venom treatments. In parallel, cytotoxicity has been established using trypan blue exclusion assay. The venom induced significant and dose-dependent DNA damage in peripheral blood cells, while DNA repair was observed after 24 h depending on the administered dose. These findings suggest that the venom may transiently alter genomic integrity, possibly through oxidative stress-related mechanisms. Characterizing the venom’s components involved in DNA damage could contribute to improved snakebite management.

## 1. Introduction

Ophidian envenomation remains a significant public health, social and economic concern, particularly in impoverished communities across intertropical and subtropical areas around Africa, Asia and Latin America [1,2,3]. In these areas, limited access to appropriate healthcare exacerbates the risk of severe complications and even death [4,5,6]. In 2017, the world health organization (WHO) recognized this pathology as a serious neglected tropical disease [7,8,9]. Each year, it is estimated that between 4.5 and 5.4 million snake envenomation’s occur worldwide; among these, approximately 1.8 to 2.7 million develop clinical manifestations, 125,000 human deaths and around 400,000 individuals suffer from permanent sequelae such as reduced mobility, amputations, blindness, or post-traumatic stress disorder [1,8,10]. The African continent remains vulnerable to snakebite envenomation, with 435,000 to 580,000 cases recorded each year, representing 20% of the total global burden [4,11,12]. In Morocco, around 300 snakebite cases are reported annually, corresponding to an incidence rate of 0.2 per 100,000 inhabitants and a case fatality rate of 4% [13,14,15]. These envenoming accidents are mainly attributed to two families of venomous snakes: The *Elapidae,* represented by *Naja haje*, which causes a neurotoxic syndrome, and the *Viperidae* represented by: *Vipera monticola*, *Vipera latasti*, *Echis pyramidum*, *Cerastes vipera*, *Bitis arietans*, *Daboia mauritanica* and *Cerastes cerastes* (*C. cerastes*), responsible for viperine syndrome that can endanger vital and functional prognosis [13,15,16].

Clinical effects of viper envenomation, including that of *C. cerastes*, encompass a broad range of local and systemic pathologies, such as inflammation (pain and edema), neurotoxicity, myotoxicity, and necrosis. These pathophysiological manifestations result from tissue and cellular damage associated with degradation in the genetic material: DNA [17,18]. The venom proteomic analysis of Moroccan *C. cerastes* reveal a relatively low complexity proteome composed of 25–30 toxins grouped into 6 major protein families, with snake venom metalloproteinases I-III (SVMPs) representing the most abundant family (61%), followed by phospholipases A_2_ (PLA_2_) (19%), dimeric disintegrins (8,5%), and snake venom serine proteinases (SVSPs) (7%). Cysteine-rich secretory proteins and C-type lectin-like proteins, each contribute less than 4% of the total venom composition. This proteomic profile underlies the characteristic pathophysiological effects of envenomation, with SVMPs type I-III being primarily responsible for coagulopathy and local hemorrhage [14,19,20]. In addition, PLA_2_s induce cytotoxicity by disrupting cell membrane integrity, promoting inflammation and inducing myonecrosis [21,22]. These enzymes are also known to trigger the excessive production of reactive oxygen species, contributing to oxidative stress and subsequent cellular and molecular impairments, including DNA damage [23,24,25]. Understanding the molecular composition of *C. cerastes* venom provides a critical foundation for investigating its downstream cellular effects, particularly oxidative stress and genotoxicity.

In this work, our overarching aim was to contribute to the initial characterization of the genotoxic and cytotoxic potential of snake venoms. Owing to the availability of *C. cerastes* venom in laboratory and its medical relevance in Morocco, we selected this venom as our experimental model. Specifically, we sought to highlight its genotoxic effects both in vitro and in vivo venom treatments using the alkaline comet assay complemented by automated image analysis of immunofluorescence microscopy images.

## 2. Materials and Methods

### 2.1. Materials

#### 2.1.1. Animals

This study was designed to evaluate the genotoxicity of *C. cerastes* venom in 76 male *NMRI* (Naval Medical Research Institute) mice. It was maintained at the Experimental Center of Pasteur Institute of Morocco, according to Good Manufacturing Practices guidelines on animal experimentation [26].

#### 2.1.2. Venom Preparation

The venom was extracted by manually stimulating the venom glands of multiple *C. cerastes* specimen’s viper at the Experimental Center of Pasteur Institute of Morocco. Subsequently, the crude venom was reconstituted in Millipore-Quality-purified (Milli-Q) water and centrifuged at 13,000× *g* for 15 min at 4 °C to remove impurities. The lyophilized supernatant of the venom obtained was stored at −20 °C until further use. Venom’s proteins were quantified by Bradford Assay [27,28,29].

### 2.2. Methods

#### 2.2.1. Electrophoretic Analysis of Venom

The electrophoretic characterization of *C. cerastes* venom proteins was performed using Sodium Dodecyl Sulfate-Polyacrylamide Gel Electrophoresis (SDS-PAGE) (Hoefer SE 260 Mighty Small II, Hoefer, Holliston, MA, USA) under denatured, reducing and non-reducing conditions, which separates proteins based on their molecular weight [30,31,32].

First, for each condition, resolving gels at 12% and 15% were prepared between two glass plates and subsequently, stacking gels at 5% acrylamide, containing the wells for protein loading, were prepared and layered on top of the polymerized resolving gels. Well L1 contained 5 µL of the protein standard solution (Tricolor Broad Protein Ladder: 3.5–245 kDa). *C. cerastes* venom was prepared at 5 µL of three protein amounts of 10, 15, and 20 µg, corresponding to wells L2, L3, and L4, respectively. To each of these venom samples, a loading buffer with/without the reducing agent β-mercaptoethanol (which breaks disulfide bonds) was added. The migration of the prepared samples was carried out using a mini electrophoresis system at a constant voltage of 7 V/cm of gel for 45 min. After migration, the gels were stained overnight at room temperature with a Coomassie Brilliant Blue staining solution. Finally, the protein bands of both the molecular weight markers and the venom samples were fixed using a methanol–acetic acid solution (Table 1).

#### 2.2.2. Determination of Median Lethal Dose (LD_50_) of Venom

The median lethal dose (LD50) of *C. cerastes* venom was determined using male NMRI mice (18–22 g). 7 groups of four mice were used. Six groups received increasing doses of venom (10-20-30-40-60-80 µg/mouse), while the control group was administered with physiological saline solution (0.9% NaCl). All injections were performed via the intraperitoneal route with a consistent volume of 500 µL/mice. During the experiment, the clinical signs of viper envenomation to be observed include: hemorrhage, neurological disorders such as agitation, shivering, paralysis of the lower limbs, miosis, hunchback posture, as well as cardio-respiratory disturbances.

Mortality was monitored over a 48 h period. The LD50 value was evaluated using the dose–response analysis module of GraphPad Prism (version 10.4.2), following WHO recommendations [26].

#### 2.2.3. Determination of Sublethal Dose of Venom

Different doses of venom below the LD_50_ were administered in mice, in order to determine corresponding sub-lethal dose (sLD). It is defined as the minimum dose capable of inducing clinical signs of envenomation without causing death. The saline solution (NaCl 0.9%) was administered to the control group. All injections were made by IP route at 500 µL/mice.

#### 2.2.4. Genotoxicity Evaluation of *Cerastes cerastes* Venom Using Alkaline Single-Cell Gel Electrophoresis (Comet Assay) Following Both In Vitro and In Vivo Venom Treatments

##### In Vitro *Cerastes cerastes* Venom Treatment

Blood samples from 2 groups of 4 mice were collected in heparinized tubes to enable analysis of the overall peripheral cellular fraction. Blood from the first group was treated with 1 LD_50_/mouse equivalent dose of *C. cerastes* venom, while the second group served as a control receiving physiological saline solution (0.9% NaCl). Comet assays were performed after 1 and 2 h of incubation to allow interaction between the venom and blood components and to assess the kinetics of venom induced DNA damage [33,34,35,36,37,38].

##### In Vivo *Cerastes cerastes* Venom Treatment

It involved 3 groups of 4 mice, each receiving IP injections of *C. cerastes* venom at 0.5 LD_50_/mouse dose and 1 LD_50_/mouse dose. Control mice treated with saline solution (0.9% NaCl). The overall peripheral cellular fraction of blood samples for comet assay were collected 6 h and 24 h post-injection [35,37].

##### Comet Assay Analysis

The comet assay procedure, based on single-cell gel electrophoresis, involved preparing immunofluorescent microscopy slides pre-coated with Low Melting Agarose (NMA). 10 µL of each blood sample containing peripheral cellular fraction, were mixed with 70 µL of Low Melting Agarose (LMA), layered onto the slides, and solidified at 4 °C for 5 min. Cells were then lysed in lysis solution (100 mM EDTA, 10 mM Tris, 2.5 M NaCl, 1% Triton X-100, and 10% DMSO, pH 10) for 1 h at 4 °C, followed by a 30-min incubation in TBE (Tampon Tris-Borate-EDTA) buffer (pH 8.3) in an electrophoresis tank at 4 °C. Afterwards, the slides were subjected to horizontal electrophoresis at a current of 300 mA (0.8 V/cm) for 20 min. Finally, slides were stained with ethidium bromide solution, and DNA morphology was examined using an Olympus fluorescence microscope (Olympus Corporation, Hachioji, Tokyo, Japan) equipped with a TRITC filter (excitation: 520 nm/emission: 590 nm).

The genotoxicity of *C. cerastes* venom was evaluated using the comet assay after in vitro and in vivo venom treatments. Therefore, DNA from blood cells was analyzed using the comet assay. Images obtained were analyzed using *OpenComet* software plugin (v1.3.1), integrated into ImageJ (version 1.50i). It evaluates the percentage of DNA in the comet head (an indicator of total DNA integrity) and the percentage in the comet tail (an indicator of DNA fragmentation) along its entire length, reflecting the extent of damage [37,39,40,41]. These data were accompanied by comparative statistical graphs. We first explored this activity using an initial in vitro genotoxicity performed at a single dose in order to highlight alterations in genetic material (DNA). This investigation was complemented, in a second step, by an in vivo study using 2 different venom doses.

#### 2.2.5. Cytotoxicity Assay

Cell death was assessed using the trypan blue exclusion assay according to Kamiloglu et al. [42], and Warren Strober [43]. It is based on the principle that viable cells possess intact plasma membranes that exclude the dye, whereas non-viable cells with compromised membrane integrity allow trypan blue to penetrate and stain the cytoplasm blue. The in vitro group was exposed to *C. cerastes* venom 1 LD_50_ for 1 h and 2 h, while in vivo groups were treated with 0.5 LD_50_ and 1 LD_50_ sampled at 6 h or 24 h. Control mice were performed with saline solution (NaCl 0.9%). Briefly, blood cell suspensions of all mice treated were mixed with an equal volume of 0.4% trypan blue solution and incubated for 2–3 min at room temperature. Viable (unstained) and non-viable (blue-stained) cells were counted using a Neubauer hemocytometer (Paul Marienfeld GmbH & Co. KG, Lauda-Königshofen, Germany) under Olympus light microscopy (Olympus Corporation, Hachioji, Tokyo, Japan), counting a minimum of 200 cells per sample in duplicate. Cell viability was calculated as: Viability (%) = (unstained cells/total cells counted) × 100, and cytotoxicity as the complementary percentage of stained cells.

#### 2.2.6. Ethics Statement

This study was conducted in accordance with the ethical principles established by the World Health Organization (WHO). All experimental procedures were reviewed and approved by the appropriate institutional ethics committee. Ethical approval was granted by the Animal Research and Biodiversity Ethics Committee (ECARB) of Hassan II University of Casablanca, under approval number CERAB2025-UH2C-EXP-052V1 (2025-03-05). All methods were performed in compliance with relevant institutional and international guidelines and regulations.

#### 2.2.7. Statistical Analysis

All results are presented as means ± standard deviation. Statistical analysis was performed using *ANOVA* with *GraphPad Prism* software (version 10.4.2). A *p*-value < 0.05 was considered statistically significant. Levels of significance are indicated as follows: * *p* < 0.05; ** *p* < 0.01; *** *p* < 0.001; **** *p* < 0.0001.

## 3. Results

### 3.1. Electrophoresis Examination

**Figure 1:**
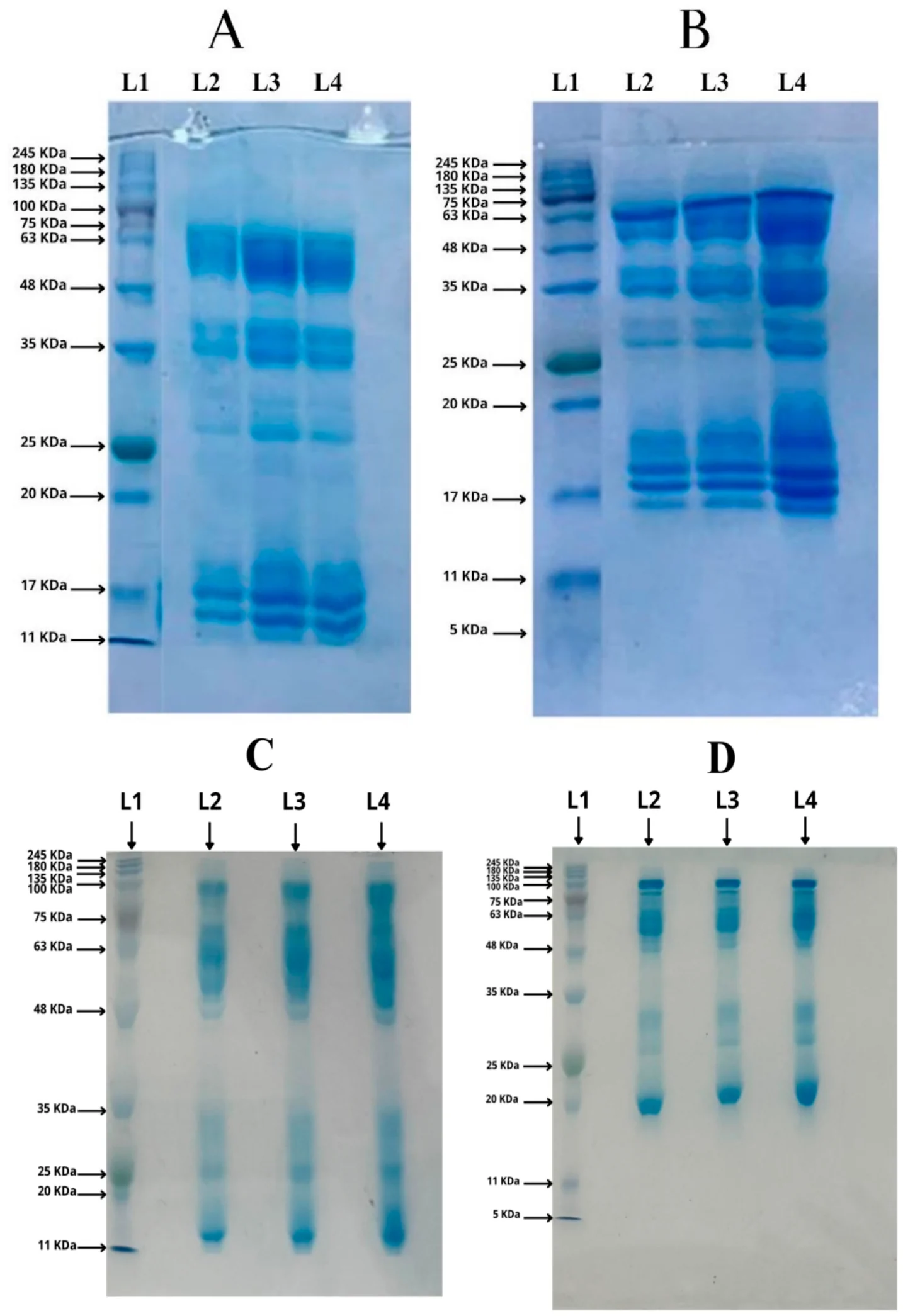
Electrophoretic profile (SDS-PAGE) of *Cerastes cerastes* venom on reducing conditions (**A**,**B**) and non-reducing conditions (**C**,**D**). (**A**,**C**) 12% polyacrylamide gel (**B**,**D**) 15% polyacrylamide gel. L1: protein molecular weight marker (5–245 kDa); L2-L3-L4: 10-15-20 µg of venom respectively.

Electrophoretic analysis of *C. cerastes* venom protein profile revealed a complex and heterogeneous composition, characterized by a wide range of molecular weights (Figure 1). Protein’s migration performed on 12% and 15% acrylamide gels showed bands of medium-intensity between 48 and 135 kDa, indicating the presence of high-molecular-weight components, corresponding mainly to snake venom metalloproteinases (SVMPs) [32]. This protein fraction is highly concentrated under non-reducing conditions (Figure 1C,D). Proteins of medium intensity were also observed between 17 and 35 kDa, primarily representing snake venom serine proteinases (SVSPs) (Figure 1) [32]. Other low-molecular-weight bands (≤17 kDa) were detected, corresponding to phospholipases A_2_ (PLA_2_) and dimeric desintegrins [32]. Overall, these findings highlight the proteomic diversity of *C. cerastes* venom and suggest the presence of various families of bioactive protein molecules.

### 3.2. Evaluation of Median Lethal Dose (LD_50_) and Sublethal Dose of Cerastes cerastes Venom

**Figure 2:**
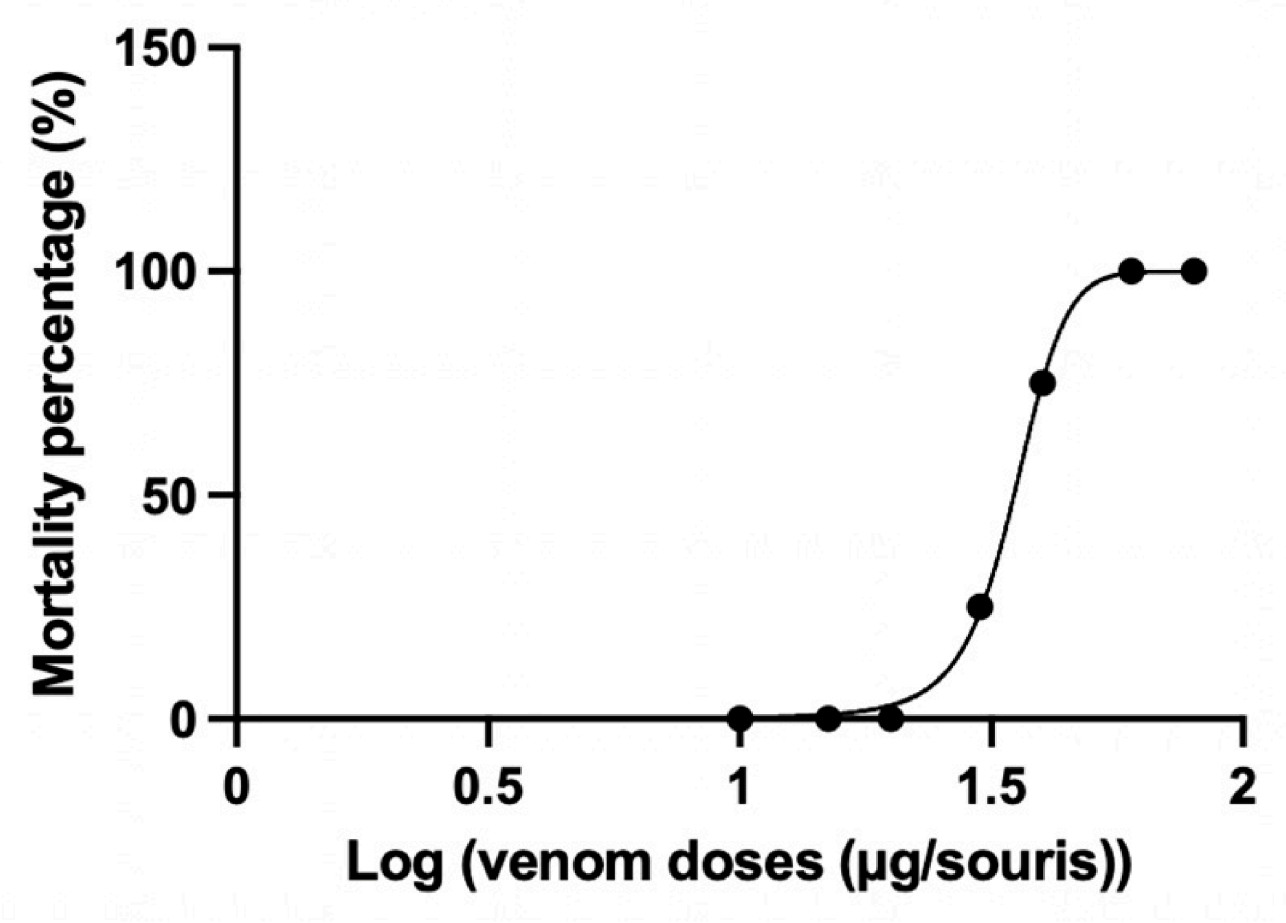
Dose–response curve of *Cerastes cerastes* venom for LD_50_ determination following intraperitoneal administration, over a 48-h monitoring period, generated using GraphPad Prism.

**Figure 3:**
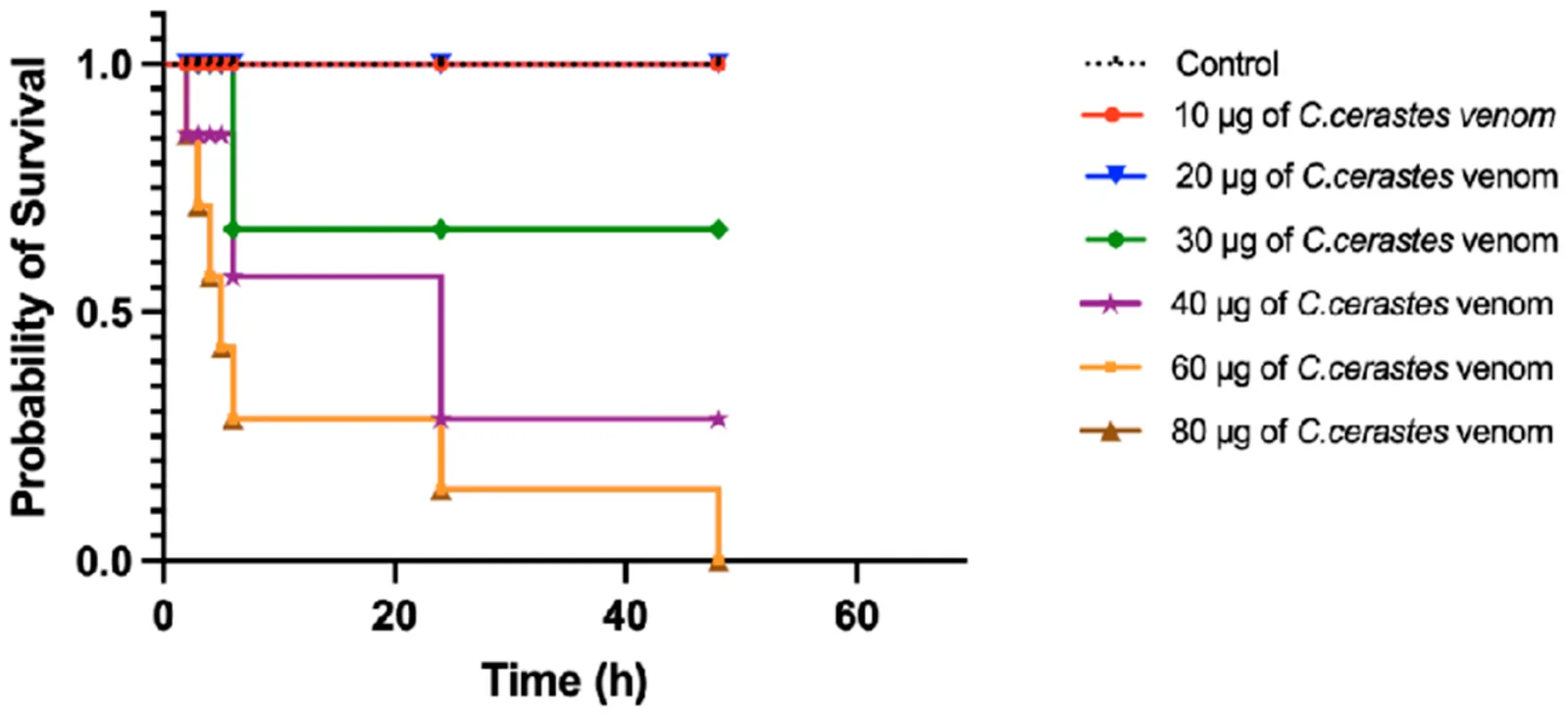
Kaplan–Meier survival curves used to support LD_50_ Determination of *Cerastes cerastes* venom. Survival was monitored for 48 h after envenomation. Curves represent the proportion of surviving mice over time for each venom dose (10, 20, 30, 40, 60, and 80 µg) and for the control group treated with 0.9% NaCl.

The LD_50_ value of *C. cerastes* venom obtained by IP injection in mice was 34.64 µg/mouse and the sublethal dose value was 20 µg/mouse (Table 2) (Figure 2). These findings indicate that *C. cerastes* venom displays a toxic potency, as even moderate doses are sufficient to induce significant pathophysiological effects, leading to death. Following envenomation, animals exhibited a range of clinical signs including itching, agitation, shivering, and epistaxis. Within 48 h after venom administration, the major pathophysiological disorders such us: cardiovascular signs included an initial tachycardia that progressively decreased and evolved into bradycardia. Pulmonary dysfunction was characterized by dyspnea, which subsequently progressed to apnea. Hemorrhagic manifestations were associated with inflammatory lesions at the injection site and an increase in the severity of tissue necrosis. Also, neurological disorders were characterized by progressive locomotor impairment, initially manifested by difficulty in movement and coordination, which gradually evolved into complete paralysis. Kaplan–Meier survival analysis revealed a dose-dependent reduction in mouse survival over the 48-h observation period, with higher venom doses resulting in earlier mortality and lower survival probabilities (Figure 3).

### 3.3. Genotoxic Effect of Cerastes cerastes Following Both In Vitro and In Vivo Venom Treatment Using the OpenComet Software

#### 3.3.1. Genotoxic Effects Associated with In Vitro Treatment with *Cerastes cerastes* Venom

**Figure 4:**
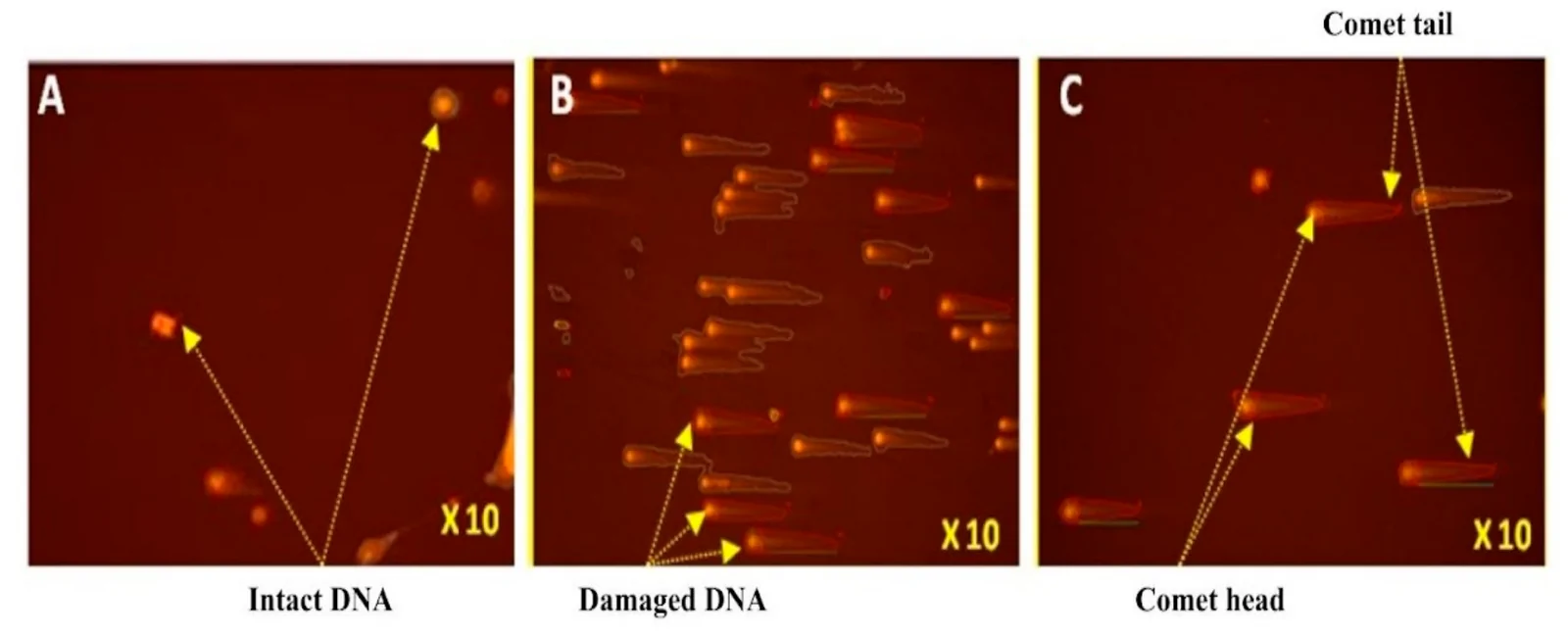
Comet assay analysis of DNA damage following in vitro treatment with *Cerastes cerastes* venom. Mouse blood was incubated with *Cerastes cerastes* venom (1 LD_50_) for 1 or 2 h. (**A**) Negative control (0.9% saline); (**B**) 1 h exposure; and (**C**) 2 h exposure.

**Figure 5:**
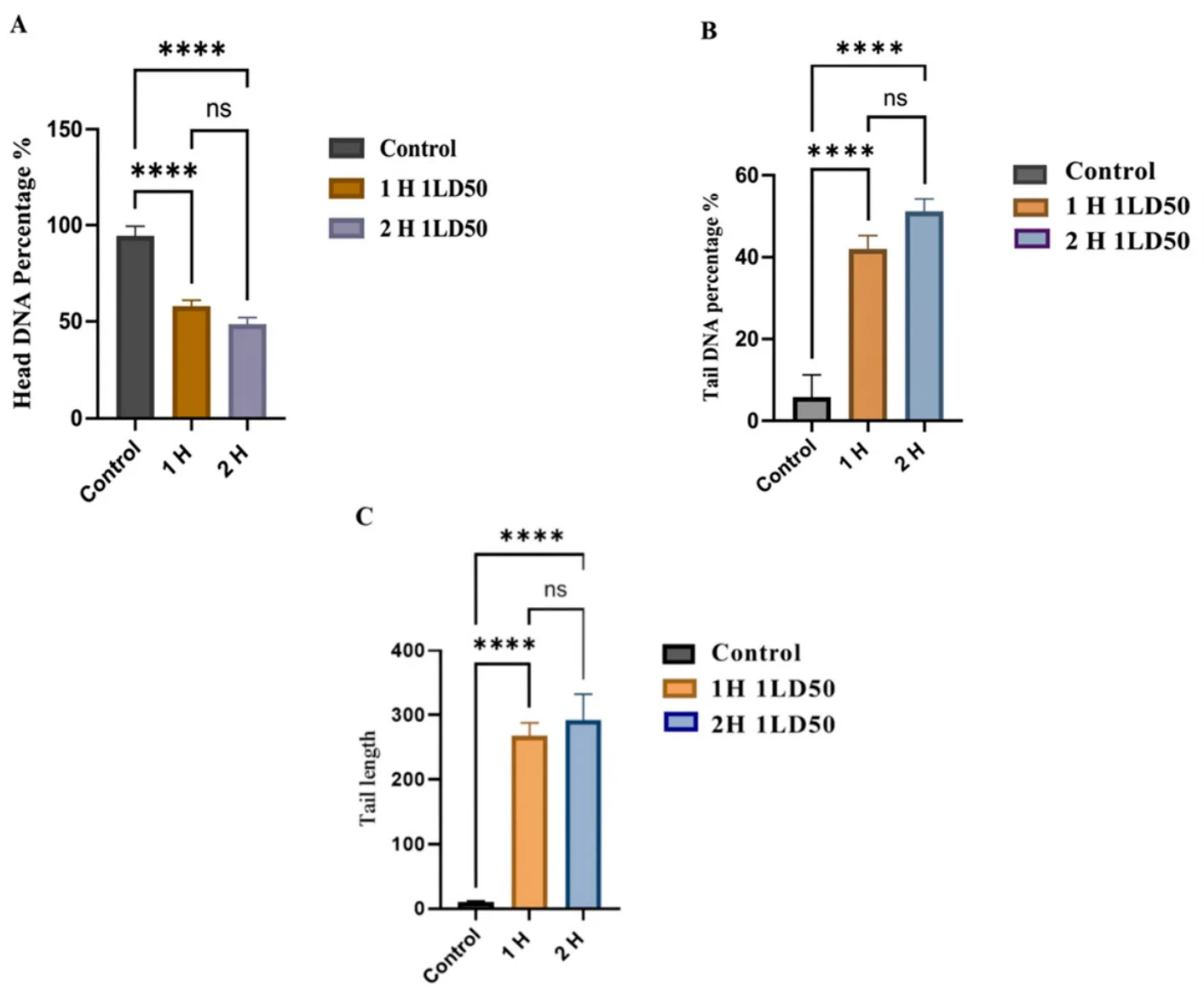
Histograms illustrating the in vitro genotoxic effect of Cerastes cerastes venom treatment at 1 LD_50_, evaluated using the comet assay. (**A**) The percentage of head DNA; (**B**) The percentage of tail DNA; (**C**) The tail length, for control and treated groups at 1 h (1H) and 2 h (2H) of incubation. Data are expressed as mean ± standard deviation (SD), with n = 4 mice per group. Statistical significance of differences between groups is indicated as follows: ns (not significant), **** *p* < 0.0001.

Blood cells from untreated mice (control group) displayed intact DNA with 100% of the signal confined to the comet head (Figure 4A). The comet assay performed on mouse blood exposed to 1 LD_50_ of *C. cerastes* venom revealed a marked loss of DNA integrity (Figure 4B,C) compared with the control group (Figure 4A). After 1 h of incubation, *OpenComet* software quantified the percentage of DNA retained in the comet head. It dropped to 55% (Figure 4B and Figure 5A), while the DNA fraction in the tail rose to 45% (Figure 4B and Figure 5B) and the tail length increased significantly (*p* < 0.0001; Figure 5C), indicating extensive DNA fragmentation. After 2 h of incubation with 1 LD_50_ of *C. cerastes* venom, a further damage aggravation of DNA was observed with reduction retained within the comet head (44%), while the DNA fraction migrating into the comet tail increased (55%) (Figure 4C and Figure 5A,B). This shift was accompanied by a more pronounced increase in tail length, reflecting enhanced DNA damage and migration after 2 h. Statistical analysis showed that the extent of DNA damage was maintained after 2 h (Figure 5). These in vitro signs of genotoxic stress were completed by in vivo setting.

#### 3.3.2. Genotoxic Effects Associated with In Vivo Treatment with *Cerastes cerastes* Venom

**Figure 6:**
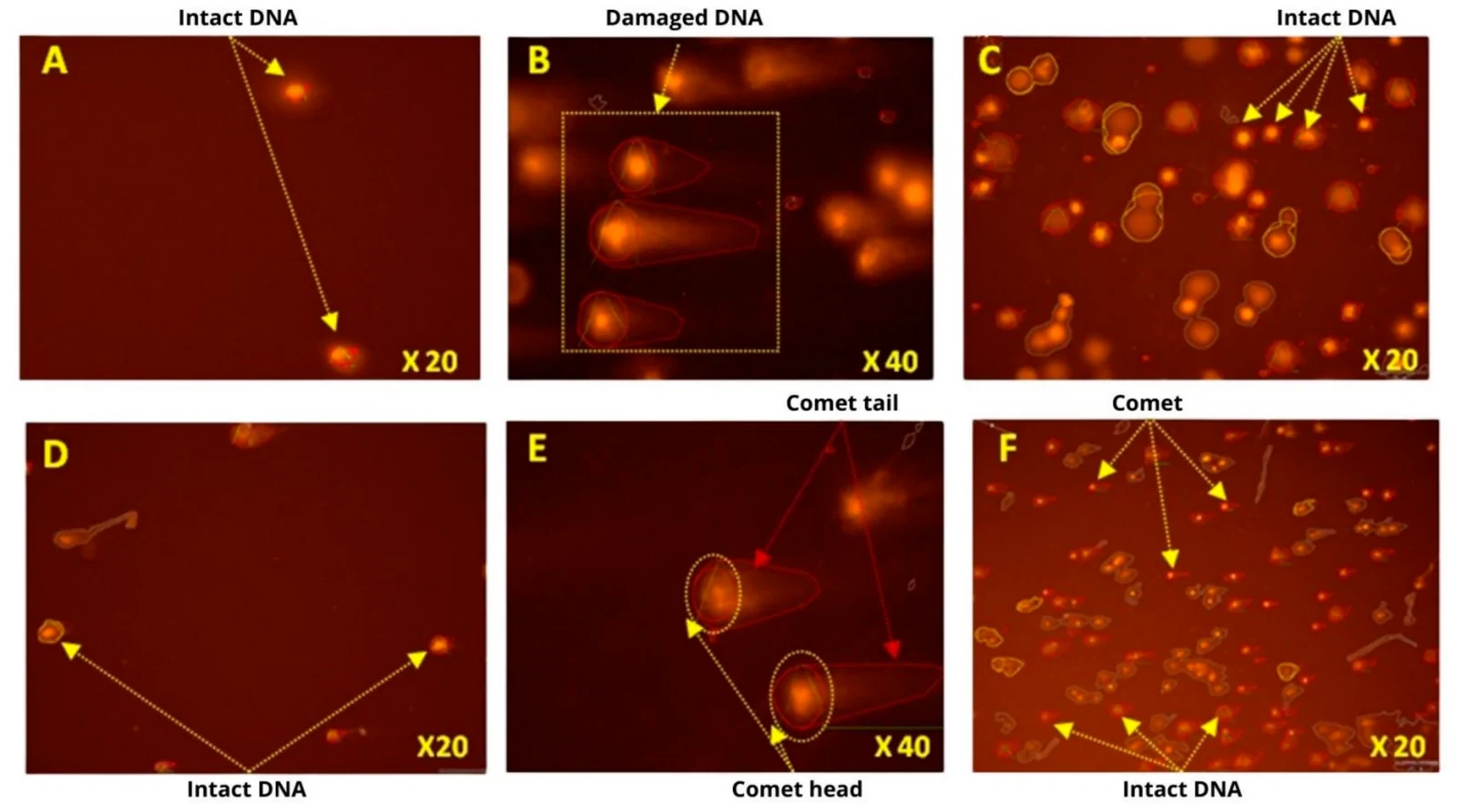
In vivo genotoxicity of *Cerastes cerastes* venom treatment in mouse blood assessed by the comet assay. Mice were injected with 2 venom doses 0.5 LD_50_ and 1 LD_50_. Blood samples were collected at 6 h and 24 h post-envenomation. (**A**,**D**) The negative control (blood treated with 0.9% NaCl); (**B**) The effects of lower dose (0.5 LD_50_) display samples taken 6 h after injection, and (**C**) samples taken 24 h after injection. (**E**,**F**) The higher dose (1 LD_50_) at the same time points (6 h and 24 h, respectively). DNA fragmentation, expressed as comet tail length and % DNA in the tail, increases with both dose and exposure time, evidencing dose-dependent in vivo genotoxic damage.

**Figure 7:**
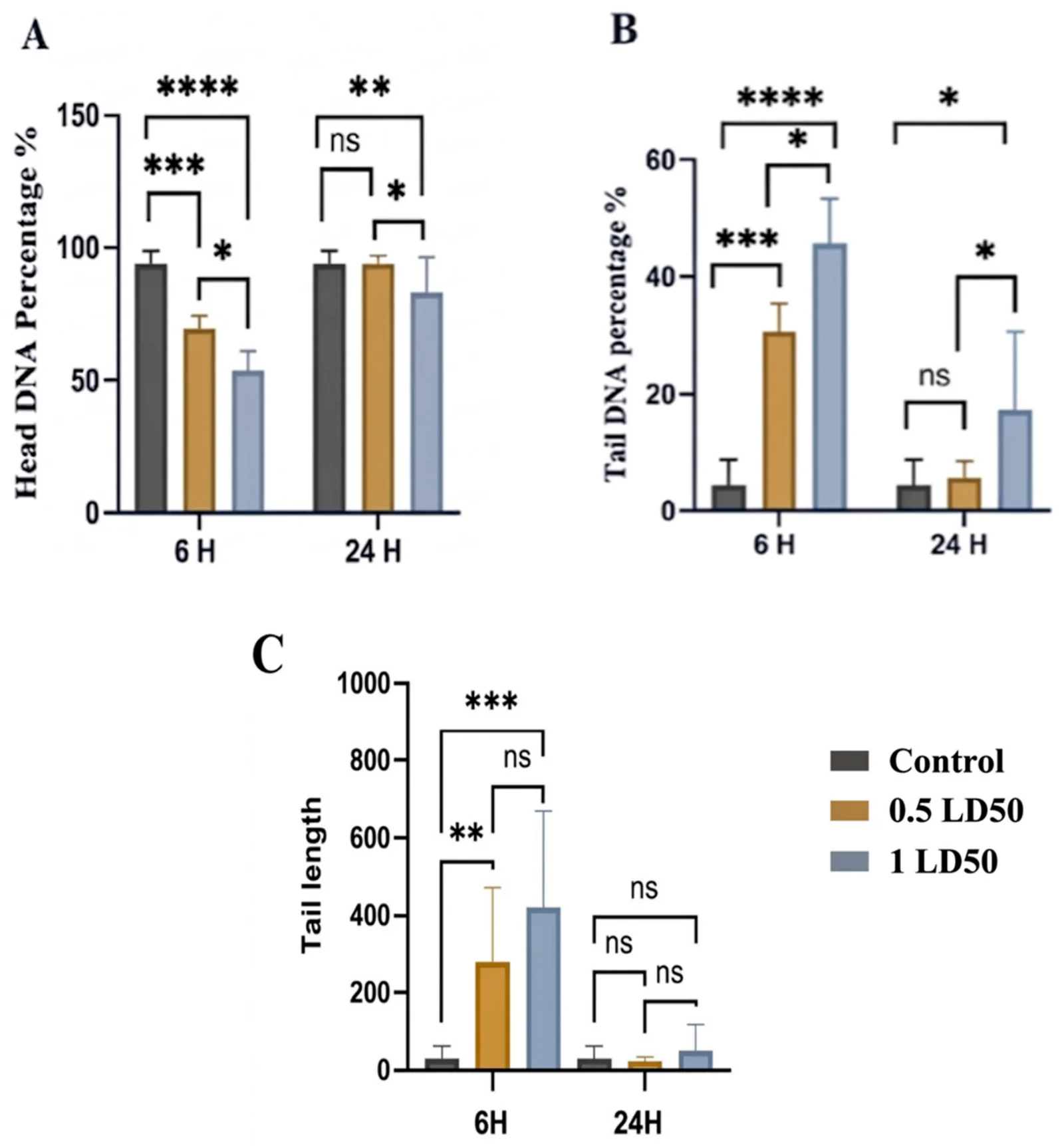
Histograms illustrating the in vivo genotoxic effect of *Cerastes cerastes* venom treatment at 0.5 LD_50_ and 1 LD_50_, evaluated using the comet assay. (**A**) The percentage of DNA in the head for the three experimental groups (control group, group treated with 0.5 LD_50_, and group treated with 1 LD_50_) at 6 h and 24 h after venom exposure. (**B**) The percentage of DNA in the tail for the same groups and exposure times. (**C**) The tail length in mice treated with 0.5 LD_50_ and 1 LD_50_ at 6 h and 24 h post-treatment. The control group received only physiological saline (0.9% NaCl). Data are expressed as mean ± standard deviation (SD), with *n* = 4 mice. Statistical significance between groups is indicated as follows: ns (not significant), * *p* < 0.05, ** *p* < 0.01, *** *p* < 0.001, **** *p* < 0.0001.

Blood cells were collected from mice envenomed with 0.5 LD_50_ and 1 LD_50_ at two kinetic posts-exposure intervals (6 and 24 h) to evaluate DNA damage using the comet assay. After 6 h of mouse exposition to 0.5 LD_50_ (20 µg per mouse), the head DNA percentage fell to 70% and the tail-DNA rose to 30%, reflecting moderate genotoxic damage (Figure 6B and Figure 7A,B). Following the 24-h interval, DNA parameters returned to initial conditions (similar to control group), suggesting complete auto-repair (Figure 6C and Figure 7A–C). At 1 LD_50_ dose (34.64 µg per mouse), a severe reduction of head-DNA to 54% was observed after 6 h, accompanied by a pronounced increase in tail DNA (46%) and tail length (Figure 6E and Figure 7A,B,D). After 24 h of mice envenoming by 1 LD_50_ venom, 20% of head DNA was fragmented showing evident recovery, with 80% head DNA and increased tail length (Figure 6F and Figure 7A,B,D). These findings indicate that increasing duration of venom exposure is associated with a reduction in DNA auto-repair capacity. *ANOVA* analysis highlights significant genotoxicity in terms of dose (0.5 LD_50_ and 1 LD_50_) and duration of toxic exposure (6 and 24 h) (Figure 7).

All results of DNA damage using comet assay approach were conducted following [35,36].

### 3.4. Cytotoxicity

**Figure 8:**
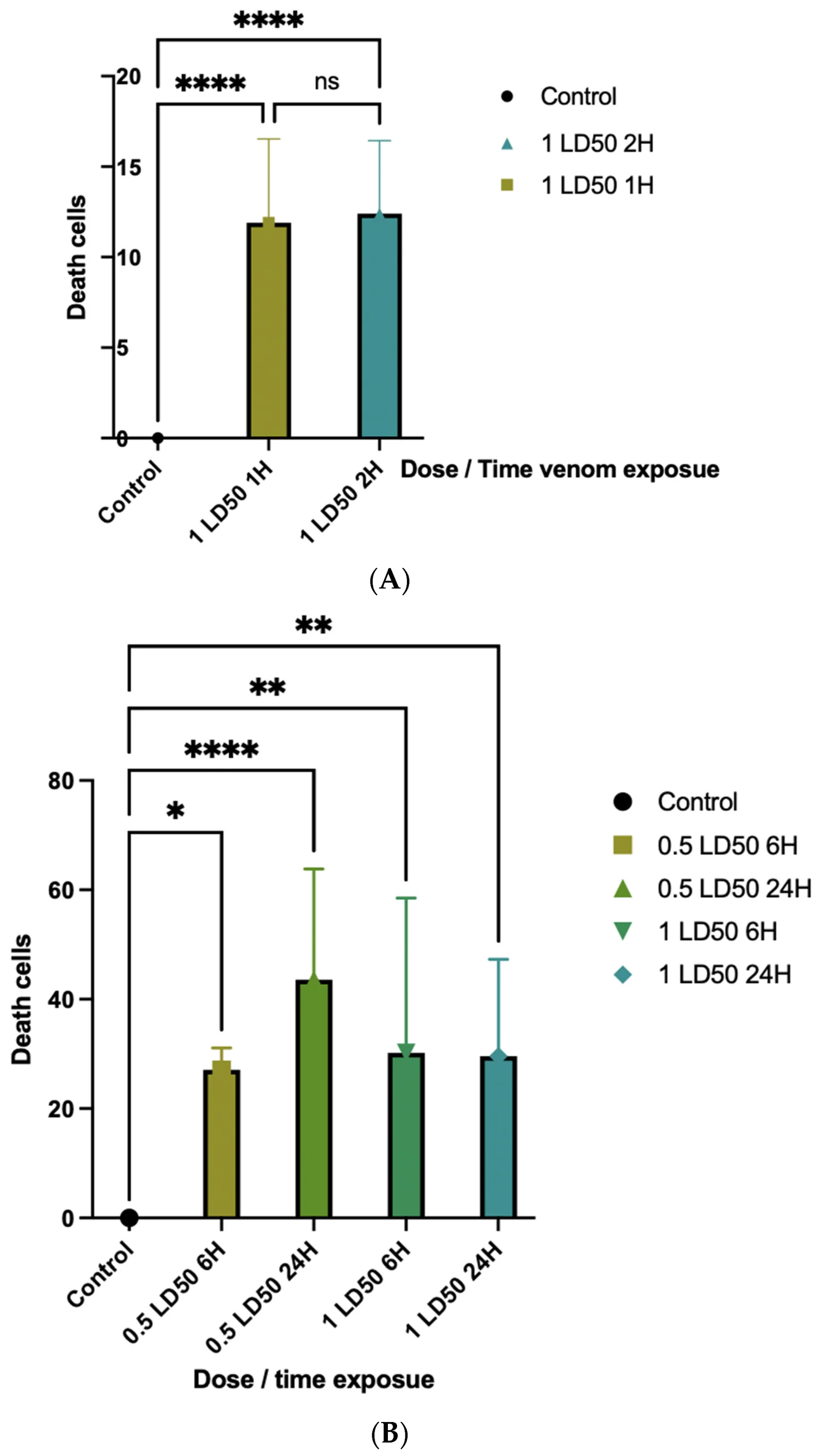
Cytotoxicity induced by *Cerastes cerastes* venom treatment evaluated by the trypan blue exclusion assay. (**A**) In vitro cytotoxicity following exposure of blood cells to 1 LD_50_ of *Cerastes cerastes* venom for 1 h and 2 h. Both exposure times significantly increased the percentage of dead cells compared with the control group (**** *p* < 0.0001), (ns) no significant difference was observed between 1 h and 2 h. (**B**) In vivo cytotoxicity after intraperitoneal administration of 0.5 LD_50_ or 1 LD_50_ of venom, with blood samples collected after 6 h and 24 h. All venom-treated groups showed a significant increase in cell death compared with the control group receiving only physiological saline (0.9% NaCl), with the highest cytotoxicity observed in the 0.5 LD_50_–24 h group. Data are presented as mean ± SD. Significance levels are indicated as ns, not significant, * *p* < 0.05, ** *p* < 0.01, and **** *p* < 0.0001.

The trypan blue exclusion assay showed 100% viable cells in the control group. Following in vitro treatment with *C. cerastes* venom, cytotoxicity increased to 22% after 1 h and 26% after 2 h at 1 LD_50_. In the in vivo experiments, cytotoxicity reached 29% at 6 h and 42% at 24 h following administration of 0.5 LD_50_ venom. Mice treated with 1 LD_50_ exhibited a cytotoxicity of 33% at both 6 h and 24 h. Overall, cytotoxicity was more pronounced in the in vivo than in the in vitro experiment and varied according to venom dose and exposure time (Figure 8).

According to Azqueta et al. [44], cytotoxicity thresholds reported for comet assay interpretation range from 20% to 50% across different studies, and no universally accepted cutoff has been established; therefore, cytotoxicity should be interpreted on a case-by-case basis.

## 4. Discussion

Snakebite envenomation represents a significant public health problem in tropical and subtropical areas, associated with severe local and systemic pathophysiological consequences. One of the most serious local outcomes is tissue necrosis, defined as uncontrolled cell death. It can be attributed to extensive DNA damage [45,46,47]. Clinically severe local necrosis from snake envenoming, particularly viper and cobra bites, can progress to tissue loss that requires partial or complete limb amputation, as shown in the Nigerian series where 16 patients (median age 12 years) lost an upper or lower limb [46]. Numerous studies have demonstrated that venom cytotoxins capable of disrupting plasma membrane integrity and cellular metabolism can also target nuclear material, leading to venom induced genotoxicity [48].

The genotoxic activity of venoms from snakes belonging to the *Viperidae* family, including those of *Crotalus* and *Bothrops* species, have been extensively investigated [38,49,50,51,52]. Also, the *Elapidae* family such as *Naja naja* and *Naja ashei* [37,53,54] and the *Colubridae* family such as *Philodryas patagoniensis* have been established [55]. However, to date, no studies have been reported on the genotoxic effect of snake venoms from Morocco. In this context, we conducted an experimental study to evaluate the in vitro and in vivo genotoxic activity of *C. cerastes* venom treatment, in murine models (NMRI mice). *C. cerastes* was selected due to the availability of its venom for controlled laboratory experimentation.

Using the alkaline comet assay, in accordance with Singh’s protocol [35,39], we evaluated the genotoxic activity of *C. cerastes* venom in peripheral blood cells. *OpenComet* software, integrated into *ImageJ*, was used to analyze microscopic images obtained after cell fixation on a slide using a single cell gel electrophoresis, allowing for the automatic measurement of parameters such as the percentage of DNA in the tail, the percentage of DNA in the head, and the tail length. This approach made it possible to identify DNA damage caused by snake venom [35,36].

The results showed significant alteration of blood cell DNA both in vitro and in vivo venom treatments. After 1 h of in vitro incubation of blood with a single dose of 1 LD_50_, DNA damage increased markedly and remained constant after 2 h. In the in vivo experiments, the injury was dose-dependent using 2 doses of *C. cerastes* venom: 0.5 LD_50_ and 1LD_50_; mice receiving 1 LD_50_ (34.64 µg/mouse) displayed more pronounced damage than those receiving 0.5 LD_50_ (20 µg/mouse). After 6 h, DNA damage was significant for both doses; however, by 24 h, moderate repair was observed for the 1 LD_50_ group, whereas complete repair was inferred for the 0.5 LD_50_ group.

The pronounced in vitro DNA damage induced by *C. cerastes* viper venom treatment is consistent with previous studies describing the genotoxic effects of venoms from the *Viperidae* family. Several studies, including that of Marcussi et al. [38] and Marcussi et al. [46], showed that crude venoms from *Crotalus durissus terrificus*, *Bothrops brazili*, *Bothrops jararacussu* and *Bothrops atrox*, when applied to human lymphocytes (in vitro study), induce a significant increase in DNA strand breaks ~5 fold compared with negative controls in the alkaline comet assay. Similarly, Moridikia et al. [56] reported that exposure of HepG2 hepatocellular carcinoma cells to *Vipera latifii* venom revealed significant DNA damage, as evidenced by increased DNA migration in the comet assay. Also, the study of Cardoso Trento et al. [57] showed that treatment of human peripheral-blood lymphocytes with crude venom from *Lachesis muta muta* induced notable DNA fragmentation, as assessed by alkaline comet assay.

Globally, these studies, all performed using cultured cell models and alkaline comet assay-based methodologies, demonstrate that snake venoms exhibit direct genotoxic activity preceding the isolation of individual venom toxins. However isolated snake venom toxins can directly induce DNA damage in vitro. *Bothropstoxin*-I from *Bothrops jararacussu* caused dose-dependent strand breaks in Human Umbilical Vein Endothelial Cells (HUVEC) and DU-145 cells [58], while crotoxin subunits from *Crotalus durissus* induced DNA fragmentation in human lymphocytes [49]. Vipoxin from *Vipera ammodytes* and its acidic component generated double-strand breaks in HepG2 cells [59]. Other toxins, such as BjussuLAAOII from *Bothrops jararacussu* similarly produced single- and double-strand DNA breaks, often linked to oxidative damage [51]. In addition to viperid venoms, genotoxic effects have also been reported for other snake families, as venom from *Philodryas patagoniensis* (*Colubridae*) induced dose-dependent DNA strand breaks in human peripheral blood mononuclear cells [55].

Consistently, in the present work the genotoxicity of *C. cerastes* venom was confirmed in murine peripheral blood cells, with severity depending on venom dose and kinetic exposure time followed by evidence of DNA repair. These in vivo investigations were aligned with those of Novak Zobiole et al. [50] reporting that the DNA damage was significantly higher in animal groups, when treated with different doses (10, 30, and 80 µg/animal) of *Bothrops moojeni* viper venom, in comparison with negative control group. Parmeswari [53] and Prabhakaran [37] have investigated the dose and time dependent genotoxic effect of *Naja naja* in *albinos* rats using alkaline comet assay. So far, only our genotoxic study has demonstrated a regenerative effect on damaged DNA.

Snake venoms exert their effects through a complex cascade beginning with direct cytotoxic damage to target cells, which subsequently progresses to genotoxic alterations. In vivo and in vitro studies have documented the cytotoxic effects of numerous snake venoms from the *Viperidae* and *Elapidae* families, highlighting substantial variability, with *Viperidae* venoms generally displaying greater cellular toxicity. This variability in cytotoxicity is strongly associated with differences in venom composition across snake families [60,61,62,63].

Toxic proteins such as SVMPs and PLA_2_, contained in *C. cerastes* venom, can cause cellular injury through various mechanisms, such as disruption of cell membranes, protein degradation, and alteration of intracellular processes. These mechanisms lead to the production of free radicals and oxidative stress, which play a crucial role in DNA damage. Generated reactive oxygen species (ROS) can directly attack the DNA molecules, causing breaks and mutations [49]. Elevated ROS levels are known to oxidize lipids, proteins, and nucleic acids, ultimately damaging DNA. As a result, cytotoxicity evolves into genotoxicity through mechanisms including single- and double-strand breaks, base modifications, interference with DNA repair pathways, and chromatin destabilization. Altogether, crude snake venom can induce DNA lesions as a secondary consequence of oxidative stress and membrane destabilization, linking its cytotoxic action to genotoxic outcomes [24,64,65].

Studies have shown that TNF-α and IFN-γ can have dose-dependent genotoxic effects on human lymphocytes through the accumulation of reactive oxygen species that cause DNA breaks [38,66]. Consequently, the composition of *C. cerastes* venom, rich in pro-inflammatory and pro-oxidative proteins, suggests a mechanism of DNA damage induction based on a synergy between oxidative stress and inflammation [64].

The recovery of DNA integrity observed in 24 h after envenomation, particularly in the lower dose (0.5 LD_50_) was supported by Azqueta and Collins [67]. DNA repair mechanisms were evaluated using a time course alkaline comet assay. After the exposition, cells were processed under ice-cold conditions, including lysis and electrophoresis at 4 °C, to minimize additional DNA damage and inhibit ongoing repair during sample preparation. DNA was quantified immediately after exposure at subsequent recovery times and a progressive reduction in comet parameters such as tail DNA percentage and tail length, was interpreted as evidence of active DNA repair processes. These results may be explained by the activation of endogenous DNA repair and cellular defense mechanisms. Following the genotoxic stress, cells rapidly activate DNA damage repair pathways, including base and nucleotide excision repair (BER and NER) [35,67].

Among venomous molecules, such as endonucleases enzymes (DNase type), can be responsible for the hydrolysis of DNA strands and the pathogenesis of genomic damage, although the underlying mechanism is less well characterized [68,69,70].

The present study evaluated the DNA-damaging potential of crude venom from *C. cerastes*.

Since cytotoxicity may contribute to DNA migration in the comet assay, cell viability was evaluated using the trypan blue exclusion assay to confirm whether venom-induced cell death could confound the DNA damage results. The assay revealed no cytotoxicity in the control group. After in vitro venom treatment, the percentage of death cells increased (22–26%), and was more pronounced following in vivo *treatment* (29–42%), with the highest value recorded 24 h after 0.5 LD_50_ administration. These results correlated with those obtained after comet assay analysis.

These findings are consistent with previous reports on *C. cerastes* venom. Kebir-Chelghoum and Laraba-Djebari [64] showed that the venom disrupts cellular redox homeostasis, inducing oxidative stress that reduces cell viability, in line with the higher cytotoxicity observed in vivo in our study. Cytotoxic effects have also been reported for other *Viperidae* toxins; Machado et al. [51] showed that BjussuLAAO-II, an isolated L-amino acid oxidase from *Bothrops jararacussu* venom, decreased the viability of HepG2 and HUVEC cells, supporting the broader cytotoxic potential of venom components across *Viperidae* species.

Further research should focus on identifying the venom components responsible for this genotoxic effect and clarifying their molecular mechanisms of action.

## 5. Conclusions

In conclusion, this study provides the first experimental evidence of the genotoxic and cytotoxic effect of *C. cerastes* venom from Morocco. By characterizing the electrophoretic and the toxicological parameters establishing its protein composition and its significant lethal potency. The results obtained via comet assay indicate that this venom induced significant DNA damage in mouse blood cells, both in vitro and in vivo venom treatments. Thus, this genotoxicity is dose-dependent, although the DNA repair observed after 24 h in vivo suggests that the cellular damage may be partially reversible. The trypan blue exclusion assay exhibited venom induced cytotoxicity. These findings support the hypothesis that *C. cerastes* act through mechanisms involving oxidative stress and inflammation, likely induced by enzymatic components such as PLA_2_ and SVMPs. This study contributes to a better understanding of the pathophysiology of envenomation in Morocco. Future work should aim to directly assess oxidative stress and inflammatory responses to clarify their roles in *C. cerastes* venom-induced genotoxicity. Finally, characterization of specific venom components would strengthen mechanistic understanding and improve translational relevance.

## Figures and Tables

**Table 1:** Composition of the solutions and buffers used for SDS–PAGE electrophoresis.

Solution		Composition
Stacking gel		Acrylamide–bisacrylamide, Tris-HCl buffer (pH 6.8), SDS, ammonium persulfate (APS), TEMED
Resolving gel		Acrylamide–bisacrylamide, Tris-HCl buffer (pH 8.8), SDS, ammonium persulfate (APS), TEMED
Loading buffer	Reducing conditions	Tris-HCl, SDS, glycerol, β-mercaptoethanol, bromophenol blue
	Non reducing conditions	Tris-HCl, SDS, glycerol, bromophenol blue
Running buffer		Tris base, glycine, SDS
Coomassie Brilliant Blue R-250 staining solution		Methanol (40%, *v*/*v*), acetic acid (10%, *v*/*v*), Coomassie Brilliant Blue R-250 (0.1%, *w*/*v*)
Fixing solution		Methanol (40%, *v*/*v*), acetic acid (10%, *v*/*v*)

**Table 2:** Lethal dose (LD_50_) and Sublethal dose of *Cerastes cerastes* venom in mice.

LD_50_ of *C. cerastes* Venom (µg/Mouse)	Confidence Interval (95%)	Number of LD_50_/mL of *C. cerastes* Venom	Sublethal Dose of *C. cerastes* Venom (µg/Mouse)
34.64	(34.43–34.86)	24	20 µg/mouse

## Data Availability

The original contributions presented in this study are included in the article. Further inquiries can be directed to the corresponding author.

## Abbreviations

The following abbreviations are used in this manuscript:

DNA	Deoxyribonucleic acid
LD_50_	Median lethal dose
*C. cerastes*	*Cerastes cerastes*
*WHO*	World Health Organization
SVMPs	Snake Venom Metalloproteinases
PLA_2_	Phospholipases A_2_
SDS	Sodium Dodecyl Sulfate
SDS-PAGE	Sodium Dodecyl Sulfate–Polyacrylamide Gel Electrophoresis
Tris HCL	Tris(hydroxymethyl)aminomethane hydrochloride
APS	Ammonium Persulfate
TEMED	Tetramethylethylenediamine
*NMRI*	Naval Medical Research Institute
NaCl	Sodium Chloride
sLD	Sublethal Dose
IP	Intraperitoneal
NMA	Low Melting Agarose
LMA	Low Melting Agarose
TBE	Tris-Borate-EDTA buffer
TRITC	Tetramethylrhodamine Isothiocyanate
*ANOVA*	Analysis of Variance
*p*	*p*-value
SD	Standard deviation
ROS	Reactive oxygen species
